# Fortification of Pea and Potato Protein Isolates in Oat-Based Milk Alternatives; Effects on the Sensory and Volatile Profile

**DOI:** 10.3390/foods13132075

**Published:** 2024-06-30

**Authors:** Roisin McCarron, Lisa Methven, Stephanie Grahl, Ruan Elliott, Stella Lignou

**Affiliations:** 1 Department of Food and Nutritional Sciences, University of Reading, Harry Nursten Building, Whiteknights, Reading RG6 6DZ, UK; 2 Arla Innovation Centre, Agro Food Park 19, 8200 Aarhus N, Denmark; 3 Department of Nutrition, Food and Exercise Sciences, Faculty of Health and Medical Sciences, University of Surrey Guildford, Surrey GU2 7YH, UK

**Keywords:** oats, plant based, milk, GC–MS, particle size, colour, bitterness, astringency

## Abstract

Oat-based milk alternatives (OMAs) are an important alternative to bovine milk, with prevalence of lactose intolerance, as well as soy and nut allergies limiting consumers options. However, OMAs are typically lower in protein content than both bovine milk and soy-based alternatives, with protein quality limited by low lysine levels, which can reduce protein digestibility. Addition of alternative plant proteins may increase the quantity of protein, as well as balancing the amino acid profile. However, plant-based proteins have additional sensory qualities and off-flavours, which may lead to undesirable characteristics when introduced to OMAs. This study aimed to assess the effect of pea and potato protein addition on the sensory profile, volatile profile, colour, and particle size in an OMA control product. Results demonstrated that pea protein contributed to a bitter and metallic taste, astringent aftertaste, and a significantly increased overall aroma correlated with higher levels of key volatiles. Whilst potato protein resulted in less flavour changes, it did lead to increased powdery mouthfeel and mouthcoating supported by a substantially increased particle size. Both protein fortifications led to detectable colour changes and a staler flavour. Fortification of OMA product with the pea protein led to significant sensory, volatile and physical changes, whilst the potato protein led to predominantly physical changes. Further investigation into alternative plant-based proteins is necessary to optimise sensory qualities whilst increasing protein content and the amino acid profile.

## 1. Introduction

Plant-based milk alternatives (PBMAs) have seen increased popularity over the past decade, now accounting for approximately 8% of total retail milk sales in the UK [1]. This may be partially due to the prevalence of lactose intolerance and cow’s milk allergies [2], as well as a desire to reduce environmental impact, with PBMAs being advertised to have a lower carbon footprint than dairy milk [3]. Oat-based milk alternatives (OMAs) are of particular interest, due to their low allergenicity, with nut allergies on the rise [4] as well as soy allergies [5], making them a popular alternative to many other PBMAs. OMAs have also been found to have an increased overall preference in sensory studies, with consumers placing OMAs highest in preference over rice, soy, hemp and lentil [6]. Oats may provide nutritional benefits resulting from the soluble fibre beta-glucan [7], and from compounds with antioxidant properties—avenanthramides [8] and avenacosides [9].

However, OMAs have a drawback in terms of protein quantity and quality. The overall level of protein in OMAs (typically 4.6 g kg^−1^) is much lower than both cow’s milk (typically 32.6 g kg^−1^), and soy-based milk alternatives (typically 37.8 g kg^−1^) [10]. Despite oats potentially containing a much higher protein content in relation to other cereals [11], cereal proteins are generally of lower nutritional quality in comparison to animal-derived protein, being limited in the essential amino acid lysine, [12]. Limited amino acids reduce the protein quality as assessed by the protein digestibility-corrected amino acid score (PDCAAS), which has been adopted by the FAO/WHO as the preferred method for measurement of protein value in human nutrition [13], with the PDCAA in oats being limited mainly by lysine [14]. As all amino acids are required for protein synthesis, a lack of one or more essential amino acids can compromise postprandial muscle protein synthesis [15]. Based on the recommended adult protein intake of 0.66 g/kg bodyweight/day, a source of only oat protein would fail to meet the WHO/FAO/UNU requirements for essential animo acids [16], with oat protein comprising only approximately 21% essential amino acids. Milk proteins, however, contain an improved balance of essential amino acids required by humans, and are highly digestible and bioavailable [17]. The protein digestibility score for oat proteins is shown in the literature to be just 41–51%, whilst cow’s milk protein has a score of 121% [5].

For this reason, it may be beneficial for OMAs to be fortified with alternative plant protein sources. Plant proteins can act as natural emulsifiers, to replace dairy proteins [18], and it has been found in some studies that intake of plant-based proteins may help reduce cardiovascular risk factors, and provide potential benefits in relation to obesity, weight management and metabolic syndrome [19]. However, allergens are a large restricting factor for many plant proteins, as soybean allergy may lead to severe allergic reaction and anaphylaxis in allergic individuals [20], whilst allergenic proteins in tree nuts may induce IgE-mediated hypersensitivity in up to 4.9% of people worldwide, potentially leading to serious and life-threatening reactions [21]. Pea protein and potato protein, however, have low allergenicity and added health benefits [22,23], as well as meeting the WHO/FAO/UNO amino acid requirements [15]. Moreover, it has also been hypothesised that potato and pea protein microparticles could add a supporting creamy texture to food products [24], contributing to enhanced organoleptic properties.

Pea protein is popular in the food industry, mainly due to its cost-effectiveness, as well as being generally hypoallergenic [22]. Studies have also shown pea protein to provide antioxidant, anti-hypersensitive, anti-inflammatory, cholesterol-lowering, and modulating intestinal bacteria activities [25]. Pea proteins contain enough essential amino acid content, at 30%, to meet the WHO/FAO/UNU recommended requirements (based on a recommended protein intake of 0.8 g/kg body weight per day), with well above the recommended leucine requirements [26], in which plant proteins are often deficient [27]. Pea proteins contain high levels of lysine, which can balance the deficiency in cereal-based proteins [28], as well as high levels of threonine and tryptophan [18]. Pea proteins include 18–25% of water soluble albumin and 55–65% of salt extractable globulin [18]. Pea protein can also provide beneficial functional properties, due to its solubility as well as emulsifying, foaming and gelling properties [25]. 

However, pea proteins have been shown to exhibit a “beany” off-flavour, due to the presence of lipoxygenase, with lipoxygenase-catalysed oxidation of unsaturated fatty acids leading to the formation of beany flavour volatiles [18]. Aldehyde molecules are generally associated with the beany flavour of pea proteins, with hexanal being the most frequently reported compound responsible for undesirable aromas [29]. Other off-flavours present have been described as “green” “vegetal” “hay-like” and “rancid”, and are a limiting factor for consumption [29]. Along with aldehydes, compounds such as ketones, alcohols, pyrazines and furans also contribute to the off-flavour profile of pea proteins [29].

Non-volatile compounds may also lead to perceived off-flavours in plant proteins, with isoflavones associated with bitterness, saponins with astringency, and phenolic acids associated with a metallic off-flavour [30]. Saponins develop from a secondary metabolism to contribute to plant defences in the presence stresses, with the content of saponins greater in pea samples that have been exposed to contamination with pathogens [31]. However, extracts of saponins from peas used in a sensory study, were described by panellists as bitter and astringent, demonstrating their role in pea off-flavours [32].

Pea protein isolates have also been shown to exhibit green and yellow colours, with lightness measured among the isolates to be significantly different depending on cultivar [33], suggesting selection of pea strain may be important in regard to achieving a sensory appealing colour profile.

Potato proteins are an attractive plant-derived protein source, with potatoes being an important global food group, with over 374 million metric tonnes of potato production per year [34]. Potato proteins are regarded as a waste product of starch manufacture [35], and can be obtained at large scale and inexpensively as by-products of processing of potatoes in the starch industry [23]. Their nutritional quality has been shown to be superior to most major plant proteins [35].

Potato proteins consist of soluble proteins, in which 75–85% are globulins, whilst approximately 25% of the total proteins are insoluble, and build up the potato cell wall [23]. Potato proteins can be divided into three main groups—patatin, protease inhibitors, and other proteins of high molecular weight [36]. Potato juice protein concentrate has been found to be an excellent source of lysine, as well as threonine [36].

However, the heat treatment potato proteins are subject to when intended for food purposes can affect their structure and biological activity, with a temperature of 80 °C having been shown to damage hydrophobic interactions and significantly change protein structure [23]. The solubility, thermal coagulation, and emulsifying properties of potato proteins have been shown to be affected by the pH and temperatures of processing [35]. Potato protein has been found to exhibit notable levels of n-hexanal, described as an undesirable compound in potato protein which may lead to the formation of off-flavours in the product [37]. The main aim of this study was to assess the effects of fortification with pea and potato protein at different concentrations on the sensory profile of OMAs, and identify whether this is significantly affected by the presence of off-flavours, or physiochemical issues arising from the protein addition. The secondary aim was to establish the possibility of raising the total protein level to be comparable with cow’s milk (3.4%), without compromising the quality characteristics of the product. To achieve this, an OMA product with and without addition of pea and potato proteins was assessed using a trained sensory panel, as well as instrumental analyses using gas chromatography-mass spectrometry to analyse the volatile compounds, a mastersizer to measure particle size, and a colourimeter to measure lightness and off-colours were conducted.

## 2. Methods

### 2.1. Materials

Oat flour (grade 3, gluten free) was obtained from Glebe Farm Foods limited (Huntington, UK). Pea protein isolate (80% protein; K830) was kindly supplied by K2B limited (Cambridge, UK). Potato protein isolate (93.2% protein) was purchased from Avebe group (Veendam, The Netherlands). Food-grade enzymes, alpha-amylase (HT30K) and glucoamylase (300k) were from Enzyme Supplies Limited (Oxford, UK).

Solid-phase microextraction standards were obtained from Sigma-Aldrich (Gillingham, UK): 1,2-dichlorobenzene (10 ppm in methanol) and alkane standards C_6_-C_25_ (100 μg/mL) in diethyl ester. Sodium chloride, HPLC-grade water, methanol and hexane, were obtained from Fisher Scientific (Loughborough, UK). LC–MS-grade formic acid (98-100%) and acetonitrile were purchased from Merck (Darmstadt, Germany), with standards avenanthramide A (i.e., 2p), avenanthramide B (i.e., 2f), and avenanthramide C (i.e., 2c), avenanthramide D phyproof^®^, and avenacoside A (>95%) purchased from Sigma Aldrich (Gillingham, UK).

### 2.2. Preparation and Production

**Figure 1:**
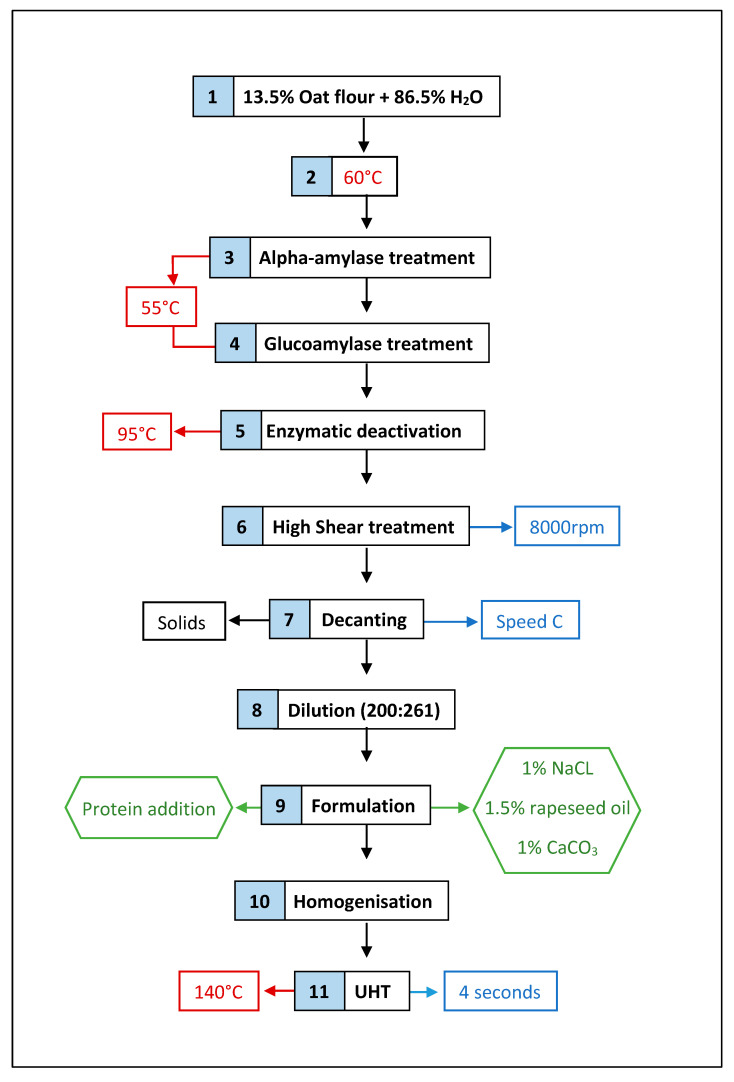
Basic processing steps used to create each OMA sample.

A control OMA product was developed (Figure 1) based on existing established methods [38], combined with optimisation of enzymatic treatment, heat setting and decanting speeds, to produce a sample with similar solid content, colour, particle size and sensory properties to existing OMAs [39].

A ratio of 13.5/86.5 oat flour to water was combined and brought up to 60 °C in a Thermomix^®^ (Vorwerk, Wuppertal, Germany), speed 2.5, with continuous stirring for five minutes, or until a visual change in consistency due to starch gelatinisation was observed. Alpha-amylase (0.5 mL) was added to 2500 g of sample and incubated for 15 min at 55 °C. As an enzyme that hydrolyses α 1-4-glycosidic linkages, breaking down starch into oligosaccharides, this enzyme is used to reduce viscosity of starch solutions. Glucoamylase (0.5 mL) was then added for a subsequent 10 min. This enzyme again hydrolyses 1,4-α-glycosidic linkages in starch, but further to produce glucose. The product was then heated to 95 °C for 5 min to deactivate enzymes, and high shear treated at 8000 rpm for 5 min. Before the decanting stage, products were allowed to cool to 70 °C, in order for decanting treatments to be consistent, preventing the risk of increased loss of solids at higher temperatures. Samples were decanted on a Westfalia separator decanter, type FRB 468518 (Oelde, Germany), at speed setting C. Due to loss of liquids via decanting and evaporation, water was added to replenish that which was lost, back to the original volume. Post decanting solid content was measured at this stage of procedure to be 13.05%, and therefore diluted accordingly back to 10%, in order to more closely match intended solid content of existing OMAs. At this stage, proteins were added and samples separated for further steps (Table 1).

The products were then heated to 70 °C in the Thermomix to hydrate the proteins, before further formulation. Rapeseed oil (1.5% *w*/*w*), salt (1% *w*/*w*), and calcium carbonate (1% *w*/*w*) were added under high shear treatment (3500 rpm). Products were then homogenised using a homogeniser (Niro Soavi, Panda, GEA Group, Germany), and underwent ultra-heat treatment, at 140 °C for a total of 4 s using an Armfield HTST/UHT system, type FT74XTS (Armfield ltd, Hampshire, England).

The protein content of oat flour was 11.7 g/100 g (Glebe Farm Foods Ltd.); therefore, at 10% (*w*/*w*) solids content, the control beverage has approximately 1% protein content. The original aim was to achieve approximately 3.4% (*w*/*w*) protein content, equivalent to bovine milk, by adding 2.4% (*w*/*w*) of either pea protein or potato protein isolate. However, samples containing this amount of added potato protein isolate caused a problem with the UHT processing, leading to overheating and a forced equipment shut-down. This may be due to aggregation, and will further be discussed below. Therefore, within the limitation of the protein isolate and equipment used in this study, the maximum addition of potato protein that could be achieved was 1% (*w*/*w*).

### 2.3. Sensory Analysis

Descriptive sensory profiling was carried out over the course of two weeks, using the trained sensory panel at the Sensory Science Centre (University of Reading), comprising eleven panellists (10 female, 1 male). All panellists had a minimum of 6 months experience and they had provided consent through their employment to taste food and use their data. During week one, three thirty minute vocabulary development and training sessions contributed to the selection of forty-five different attributes for scoring. To develop these attributes, coded samples were given to the panellists, and they were asked to describe appearance, aroma, taste, flavour, mouthfeel, and aftertaste/after-effects and produce as many descriptive terms as seemed appropriate. Reference materials (Table 2) were used for panellists to confirm if the attribute was the appropriate descriptor.

Once the consensus vocabulary was set, the panellists re-evaluated the OMAs and decided on anchors for the line scales. This led to an agreed profile of four appearance terms, 13 odour terms, 14 taste/flavour terms, four mouthfeel terms and 10 aftertaste/after-effects terms. The quantitative sensory assessment took place in isolated sensory booths, each equipped with an iPad. Compusense Cloud Software (Compusense, Guelph, ON, Canada) was used to for questionnaire and experimental design and to acquire the sensory data. The samples were provided in glass cups (50 mL), with a saucer placed over the top. This was prepared approximately five minutes in advance of each sampling to create a headspace for aroma detection. All samples were presented at 10–14 °C at a room temperature of 23 °C. The samples were randomly assigned two three-digit codes each (one for each of the two repeats) and given to the panel in a sequential balanced order. Over three days, the panel analysed each of the samples twice, and scored for each attribute using unstructured line scales (0–100). Panellists were instructed to sniff the samples first to score the aroma attributes, then assess the appearance before tasting (and swallowing) the samples to score the overall taste/flavour and mouthfeel attributes. There was a 30 s pause after the end of mouthfeel attributes and the panellists subsequently scored after-effects. Between samples, panellists cleansed their palate with water and crackers, with a 30 s pause between samples.

### 2.4. Instrumental Analysis

#### 2.4.1. Volatile Compounds—Solid-Phase Microextraction Followed by Gas Chromatography-Mass Spectrometry (SPME GC–MS)

Three millilitres of each sample were weighed into a SPME vial of 15 mL fitted with a screw cap and 0.5 g of sodium chloride was added along with 5 μL of 1,2-dichlorobenzene (10 ppm in methanol) as an internal standard and the extraction performed as described previously [39]. Volatile compounds were identified, or tentatively identified, by comparison of each mass spectrum with spectra from authentic compounds analysed in our laboratory, or from the NIST mass spectral database (NIST/EPA/NIH Mass Spectral database, 2021), or spectra published elsewhere. A spectral quality value of >80 was used alongside linear retention index (LRI) to support the identification of compounds where no authentic standards were available. LRI was calculated for each volatile compound using the retention times of a homologous series of C_6_-C_25_ *n*-alkanes and by comparing the LRI with those of authentic compounds analysed under similar conditions. The approximate quantification of volatile compounds was calculated from GC peak areas, by comparison with the peak area of the 1,2-dichlorobenzene standard, using a response factor of 1. Three replicates from each sample were analysed.

#### 2.4.2. Colour Analysis

Using a colorimeter, Konica Minolta Chroma meter CR-400, CIELAB system (illuminant C, 10⁰ viewing angle, with an 8 mm diameter port), three repeated measurements were obtained for each sample. The samples were held in a glass cell (diameter 60 mm × 15 mm) and the lightness (L*), red/green coordinate (+/−a*) and yellow/blue coordinate (+/−b*) were recorded to give a measure of the lightness and colour.

#### 2.4.3. Particle Size Analysis

A Malvern Mastersizer S was used to obtain measurements of particle size (suitable for readings above 1 μm. Three replicates were carried out, one after the other on the instrument, with the water flushed out between each reading to reduce residual particles.

### 2.5. Statistical Analysis

The quantitative data for each compound identified in the GC–MS, colour and particle size measurements were analysed by one-way analysis of variance (ANOVA) using XLSTAT Sensory (Version 2022.5.1.1388). For those compounds or physical parameters exhibiting significant difference in the one-way ANOVA, Tukey’s honest significant difference (HSD) test was applied for multiple pairwise comparisons. SENPAQ (Qi Statistics, Kent, UK) was used to carry out ANOVA and principal component analysis (PCA) using the covariance matrix, of the sensory panel data. For the sensory data, two-way ANOVA was used where the samples were fitted as fixed effects and the assessor as random effects, and both of these treatments were tested against the sample by assessor interaction. Tukey’s HSD post hoc test was applied for pairwise comparisons. In all multiple pairwise comparisons, significance was assumed at *p* ≤ 0.05. Multiple factor analysis was applied to correlate the means for the sensory data (taken over the panellists) with the means of volatile data using XLSTAT.

## 3. Results

### 3.1. Sensory Analysis

The sensory trained panel agreed to use 45 terms for the quantitative assessment of the samples. Table 3 gives the mean panel scores for these attributes and significant differences for the samples as determined by ANOVA. This table shows that 23 out of 45 attributes were significantly different between the five samples. The panellists’ individual results were analysed for repeatability and reliability. No obvious anomalies were observed as the panel generally scored to a consistent standard with one another.

Of the 13 aroma attributes (Table 3), the only significant difference was the overall intensity, where the 2.4% pea was significantly higher than all other samples except for the 1% pea. This suggests that the pea protein was responsible for imparting stronger overall aroma, although no significant difference in any specific aroma note was found.

All four appearance attributes differed significantly between samples. Notably, the off-white colour was significantly higher in both the 2.4% pea and the pea/potato combination product than the control. The 1% potato sample exhibited a significantly higher score for glass cling than the 1% pea and control samples, whilst the control had significantly more froth/foam and the highest bubble size compared to all protein-fortified samples.

Three of the four tastes differed significantly between products; the 2.4% pea was significantly less sweet and more bitter than the control. This suggests that the bitterness from the pea protein may be masking sweet-notes. This pea product was also significantly more metallic than the potato-fortified product. Of the ten flavour attributes that defined the OMAs, three differed significantly between products. The 2.4% pea had a significantly higher stale flavour than the control. All of the protein-fortified products were significantly lower than in the single cream aroma flavour than the control (although the level was low in the control also), and higher in the floury flavour.

All four mouthfeel attributes differed significantly between the products. Where potato protein was added (at 1% or in the combined protein sample) this significantly increased both mouthcoating and powdery compared to the control. These differences were not aligned with the differences in body (perception of viscosity), which was significantly lower in the 1% potato sample compared to the 2.4% pea sample. Astringency was significantly higher in the 2.4% pea sample compared to the control and the potato protein-containing products.

Four of the five aftertastes differed significantly between products, and these reflected the in-mouth differences in taste. Both of the samples containing pea (1 and 2.4%) were significantly higher in bitter aftertaste and lower in sweet aftertaste than the control. A similar pattern was found with metallic aftertaste where both pea-containing products were significantly higher than the products containing potato protein. The single cream note remained significantly higher as an aftertaste in the control product.

The significant differences in three mouthfeel aftereffects reflected the in-mouth mouthfeel differences, the 1% potato and pea/potato combination samples both had the significantly highest mouthcoating and were the most powdery, whilst the pea protein was responsible for astringent aftereffect.

### 3.2. Volatile Analyses

A total of 55 compounds were identified within the headspace of the five samples (Table 4). These included three esters, 16 aldehydes, 13 ketones, seven furans, three alkanes, four alkenes, four terpenes, and eight alcohols, as well as one alkadiene, an organosulphur, an alkylpyrazine, and an organochlorine (categorised as “other”).

The 2.4% pea was found to be significantly higher in 41 of these 55 compounds, four of which were only detected in the pea protein-fortified samples. The 1% potato was found to be significantly highest in only one compound, 2-methylthiophene, a sulphur compound. This compound was also only detected in the samples containing potato protein, suggesting that it resulted directly from the potato protein. Likewise, 2-octene, 2,3 octadiene, 2-nonanone and 2-decanone were only found in the pea protein-fortified samples, suggesting that these compounds were a direct result of the pea protein fortification of the samples. 1,3-Pentadiene, also referred to as piperylene, was substantially higher in the pea/potato combination product than all others.

### 3.3. Relating the Sensory Flavour Profile to the Volatile Composition through Multiple Factor Analysis

**Figure 2:**
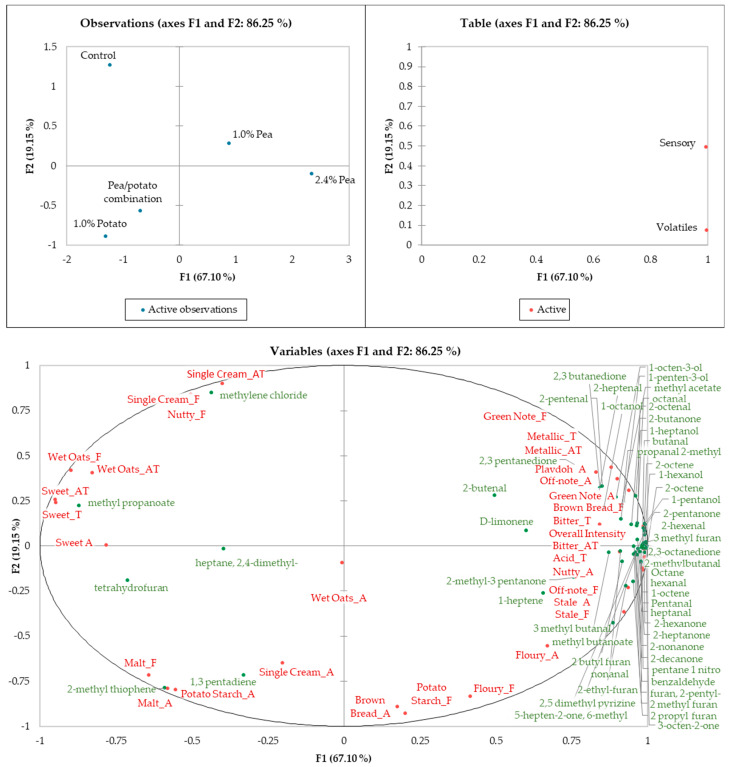
Multiple factor analysis correlating sensory data with volatile results.

Figure 2 shows that the product containing 2.4% pea protein was positively correlated with the majority of volatiles (49 out of 55), as well as with many sensory aromas and flavours, potentially resulting from these volatiles. However, the pea protein-containing products appear to be negatively correlated with sweet aromas and tastes, whilst the control product was positively correlated with single cream flavour and aftertaste, as well as nutty flavour.

**Table 4:** Volatile compounds identified in the headspace of six samples analysed by SPME GC–MS.

Compound	LRI ^a^	Confidence ^b^	Aroma Descriptor ^c^			Sample ^d^			Significance of Sample (*p* Value) ^e^
				Control	1% Pea	1% Potato	2.4% Pea	Pea/Potato Combination	
**Esters**									
methyl propanoate	628	A	Fruity, rum	2.40 ^b^	1.82 ^ab^	2.38 ^b^	1.30 ^a^	1.71 ^ab^	0.026
methyl butanoate	720	A	Fruity, creamy	6.00 ^a^	7.14 ^a^	5.79 ^a^	19.9 ^a^	5.81 ^a^	0.544
**Aldehydes**									
2-methylpropanal	552	A	Cereal/straw	9.57 ^ab^	10.5 ^b^	7.10 ^a^	15.8 ^c^	10.0 ^b^	<0.0001
3-methylbutanal	649	A	Fruity	32.7 ^a^	105.6 ^b^	83.9 ^b^	186.8 ^c^	102.1 ^b^	<0.0001
2-methylbutanal	659	A	Cocoa	16.7 ^a^	34.4 ^b^	12.9 ^a^	57.3 ^c^	23.4 ^a^	<0.0001
hexanal	802	A	Green	3767.4 ^ab^	16,083.0 ^c^	2052.6 ^a^	26937 ^d^	8141.7 ^b^	<0.0001
2-hexenal	853	A	Green	20.6 ^ab^	64.5 ^c^	12.8 ^a^	99.2 ^d^	36.5 ^b^	<0.0001
heptanal	903	A	Green	127.4 ^ab^	241.4 ^cd^	100.6 ^a^	300.1 ^d^	196.3 ^bc^	<0.0001
2-heptenal	951	A	Green	74.5 ^a^	172.2 ^b^	46.0 ^a^	146.1 ^b^	47.9 ^a^	<0.0001
benzaldehyde	959	A	Almond	60.3 ^ab^	119.1 ^c^	49.0 ^a^	244.7 ^d^	95.2 ^bc^	<0.0001
octanal	1007	A	Fruit-like	63.1 ^ab^	197.2 ^c^	40.6 ^a^	214.7 ^c^	84.7 ^b^	<0.0001
butanal	600	A	Chocolate, malty	18.9 ^a^	47.4 ^c^	11.80 ^a^	82.9 ^d^	32.3 ^b^	<0.0001
2-octenal	1063	A	Fatty	10.9 ^a^	32.0 ^b^	8.12 ^a^	35.7 ^b^	11.7 ^a^	<0.0001
2-butenal	650	A		3.23	16.1	1.60	5.16	1.85	0.122
pentanal	701	A	Fermented, bready	413.6 ^a^	1000.7 ^c^	296.2 ^a^	1200.9 ^d^	675.6 ^b^	0.006
2-pentenal	754	A	Green	23.9 ^b^	53.3 ^c^	11.1 ^a^	45.5 ^c^	19.4 ^ab^	<0.0001
nonanal	1087	B	Rose-orange	90.4 ^a^	161.0 ^b^	84.8 ^a^	163.8 ^b^	127.8 ^ab^	0.003
**Ketones/Diketones**									
2,3 butanedione	593	A	Buttery	8.82 ^a^	8.60 ^a^	7.67 ^a^	10.6 ^a^	8.02 ^a^	0.163
2-butanone	598	A	Sharp sweet	35.0 ^ab^	44.3 ^b^	26.8 ^a^	75.2 ^c^	31.9 ^ab^	<0.0001
2-methyl-3-pentanone	749	A	Mint	3.10 ^a^	3.97 ^b^	3.22 ^ab^	3.720 ^ab^	3.61 ^ab^	0.040
2-pentanone	685	A	Fruity, woody	20.2 ^ab^	43.5 ^c^	11.2 ^a^	62.6 ^d^	26.5 ^b^	<0.0001
2-hexanone	791	A	Fruity	6.72 ^a^	58.0 ^b^	3.46 ^a^	127.7 ^c^	20.7 ^a^	<0.0001
2-heptanone	893	A	Fruity	70.5 ^a^	642.6	24.3 ^a^	1640.3 ^c^	282.5 ^a^	<0.0001
3-octen-2-one	1040	A	Nutty, blue cheese	9.71 ^a^	29.9 ^b^	4.25 ^a^	44.4 ^c^	10.4 ^a^	<0.0001
2-decanone	1188	B	Orange, fermented	ND ^a^	8.59 ^c^	ND ^a^	27.3 ^d^	1.96 ^b^	<0.0001
2-nonanone	1092	A	Sweet green	ND ^a^	21.5 ^c^	ND ^a^	62.6 ^d^	7.68 ^b^	<0.0001
2,3 octanedione	985	A	Herbal	ND ^a^	25.1 ^c^	ND ^a^	36.1 ^d^	9.69 ^b^	<0.0001
2,3 pentanedione	696	A	Buttery	40.4 ^a^	51.6 ^a^	28.6 ^a^	58.0 ^a^	14.2 ^a^	0.119
6-methyl-5-hepten-2-one	986	A	Citrus, fruity	19.8 ^a^	31.9 ^b^	17.8 ^a^	43.0 ^c^	19.5 ^a^	<0.0001
**Furans**									
2-methylfuran	603	A	Chocolate	2.60 ^a^	10.6 ^b^	4.28 ^a^	20.6 ^c^	4.59 ^a^	<0.0001
3-methylfuran	611	A		0.12 ^a^	2.19 ^b^	ND ^a^	4.32 ^c^	0.74 ^a^	<0.0001
2-ethylfuran	700	A	Malty, beany	28.1 ^a^	118.1 ^a^	40.6 ^a^	174.3 ^a^	106.7 ^a^	0.081
tetrahydrofuran	626	A	Ether	86.3 ^a^	64.2 ^a^	106.9 ^a^	65.3 ^a^	67.8 ^a^	0.251
2-butyl furan	892	B	Spicy	2.78 ^a^	10.5 ^a^	3.31 ^a^	41.1 ^b^	7.40 ^a^	<0.0001
2-propyl furan	790	A		2.88 ^a^	10.5 ^b^	5.72 ^ab^	22.4 ^c^	7.66 ^ab^	<0.0001
2-pentyl furan	992	A	Fruity, green	205.8 ^a^	1244.5 ^b^	118.4 ^a^	3761.8 ^c^	642.0 ^a^	<0.0001
**Alkanes**									
octane	800	A	Gasoline	109.2 ^a^	1428.6 ^b^	49.2 ^a^	3007.5 ^c^	242.0 ^a^	<0.0001
1-nitropentane	943	B		ND ^a^	43.1 ^b^	ND ^a^	104.2 ^c^	5.58 ^a^	<0.0001
2,4 dimethyl heptane	821	B		132.3 ^ab^	102.6 ^a^	112.8 ^a^	118.0 ^ab^	152.4 ^b^	0.008
**Alkenes**									
1-octene	794	A	Gasoline	6.51 ^a^	18.1 ^bc^	6.34 ^a^	27.0 ^c^	13.6 ^ab^	<0.0001
(E)-2-octene	804	A		ND ^a^	13.6 ^b^	ND ^a^	28.9 ^c^	0.76 ^a^	<0.0001
(Z)-2-octene	811	A		ND ^a^	13.8 ^b^	ND ^a^	24.4 ^c^	2.83 ^a^	<0.0001
1-heptene	689	A		6.38	8.98	5.76	13.1	12.8	0.136
**Terpenes**									
D-limonene	1034	A	Citrus	32.1 ^b^	52.2 ^d^	41.4 ^c^	47.4 ^cd^	18.2 ^a^	<0.0001
**Alcohols**									
1-pentanol	763	A	Fermented	227.0 ^ab^	437.1 ^c^	161.7 ^a^	561.0 ^d^	255.0 ^b^	<0.0001
1-hexanol	867	A	Herbal	18.5 ^a^	50.3 ^b^	10.8 ^a^	89.3 ^c^	22.1 ^a^	<0.0001
1-heptanol	968	A	Green	19.3 ^ab^	26.2 ^bc^	14.0 ^a^	31.8 ^c^	16.9 ^ab^	0.002
1-octen-3-ol	983	A	Mushroom	82.9 ^b^	217.6 ^c^	47.8 ^a^	210.4 ^c^	44.7 ^a^	<0.0001
1-octanol	1073	A	Waxy, mushroom	19.3 ^a^	55.5 ^b^	17.1 ^a^	48.5 ^b^	11.3 ^a^	<0.0001
1-penten-3-ol	672	A	Green	49.9 ^b^	123.1 ^c^	32.0 ^a^	131.6 ^c^	27.2 ^a^	<0.0001
**Other**									
1,3 pentadiene	515	B	Acrid	ND ^a^	5.85 ^b^	38.3 ^d^	13.6 ^c^	80.5 ^e^	<0.0001
2-methylthiophene	786	A	Sulphur	ND ^a^	ND ^a^	3.30 ^c^	ND ^a^	1.83 ^b^	<0.0001
methylene chloride	531	B	Sweet	404.1 ^b^	7.09 ^a^	6.74 ^a^	7.36 ^a^	7.60 ^a^	<0.0001
2,5 dimethylpyrazine	917	A	Chocolate, musty, beefy	ND ^a^	5.84 ^b^	0.00 ^a^	16.4 ^c^	0.75 ^a^	<0.0001

^a^ Linear retention index on a HS-5MS column. ^b^ Confidence in identification of samples; A = “Identified against authentic standards in the same laboratory, B = Identified using spectra and LRI from the NIST library where the same column type had been used. ^c^ Aromas obtained from TheGoodScents company, and PubChem. ^d^ Estimated quantities (ng) collected from the headspace of 3 mL of OMA sample calculated by comparison with 20 uL of 10 ppm 1,2-dichlorobenzene used as internal standard; means (from three replicate samples) not labelled with the same letter in a row were significantly different (*p* < 0.05); as determined by Tukey’s honest significant difference (at *p* = 0.05); nd, not detected. ^e^ Significance of sample effect (*p*-value).

The potato protein-containing samples appear to be more similar to each other than other samples, whilst the pea protein products were found to be similar to one another. The potato protein samples were positively correlated with malty flavour and aroma.

Stale flavour, which was found to be significantly higher in the 2.4% pea protein-containing product compared to the control, may be associated with certain volatiles that were also detected in higher quantities in this sample. Potential associations may include 2-methylpropanal, which has been described to have a cereal/straw aroma, pentanal, described as fermented/bready, 2 pentanone which imparts a woody aroma, 3-octen-2-one with a nutty/blue cheese aroma, 1-octene and octane which are both described as having a gasoline aroma, and 2,5 dimethylpyrazine, which is described as musty. This sample also exhibited the highest amounts of hexanal, which is associated with a “beany” and “off” flavour [2], and is considered as a rancidity marker [40], which may potentially have resulted in an association with the stale aroma. However, these correlations may not be causative.

### 3.4. Particle Size

**Figure 3:**
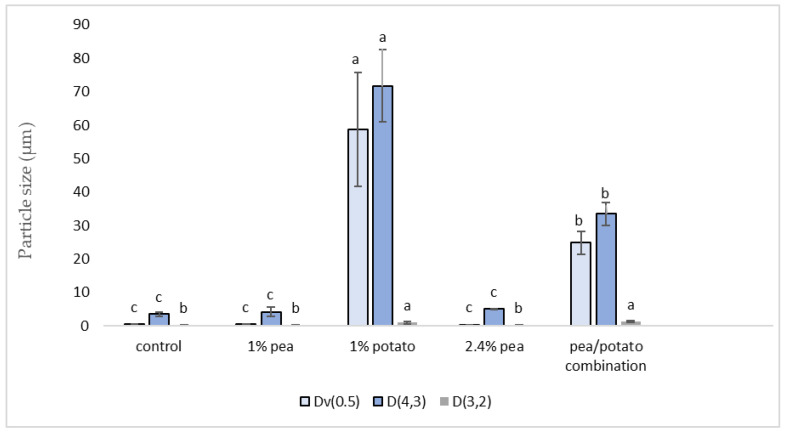
Particle size measurements: D,4,3 (volume-weighted mean/mass moment mean diameter), Dv0.5 (50th percentile volume distribution), D3.2 (surface area-weighted mean) μm. Data represent the means of three replicates ± standard deviations (*p*-value < 0.0001). Differing small letters represent sample significance from multiple comparisons as determined by Tukey’s HSD (at *p* = 0.05).

Figure 3 shows that the particle size in the product containing 1% potato protein was significantly higher than all others, with the pea/potato combination being significantly higher than the remaining three samples. This shows that the potato proteins are directly associated with large particle size. The particle size of the 1% potato sample was measured before UHT, to confirm whether this process resulted in coagulation, or whether the potato protein isolate had a larger particle size.

**Figure 4:**
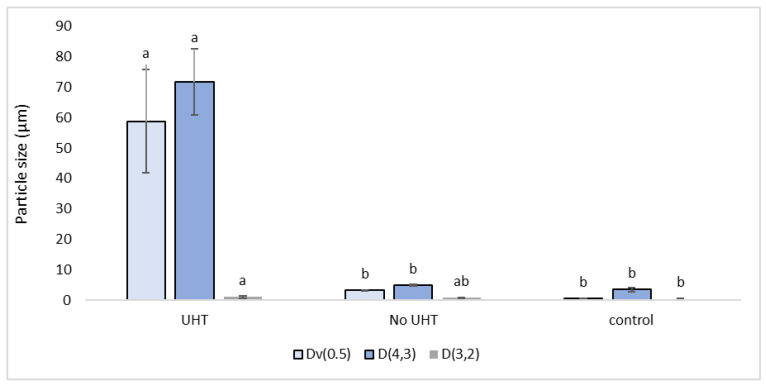
Particle size measurements: D,4,3 (volume-weighted mean/mass moment mean diameter) mm, Dv0.5 (50th percentile volume distribution), D,3.2 (surface weighted mean) μm, comparing 1% potato-fortified sample having undergone ultra-heat treatment (UHT), compared with non-ultra-heat treated 1% potato sample (No UHT), along with control product UHT treated, with no potato protein (control). Data represent the means of three replicates ± standard deviations (*p*-value < 0.0001). Differing small letters represent sample significance from multiple comparisons as determined by Tukey’s honest significance difference (at *p* = 0.05).

Figure 4 shows that the 1% potato product significantly increased in particle size after having undergone UHT. The UHT sample was significantly larger in terms of median, and volume-weighted mean diameter, than the non-UHT potato protein-fortified sample and the control. The non-UHT potato protein sample, was not significantly larger than the control in these measurements, suggesting that coagulation of potato proteins is occurring during the heat treatment. Further investigation into the mechanisms of this, and methods for prevention may be necessary.

### 3.5. Colourimeter

**Figure 5:**
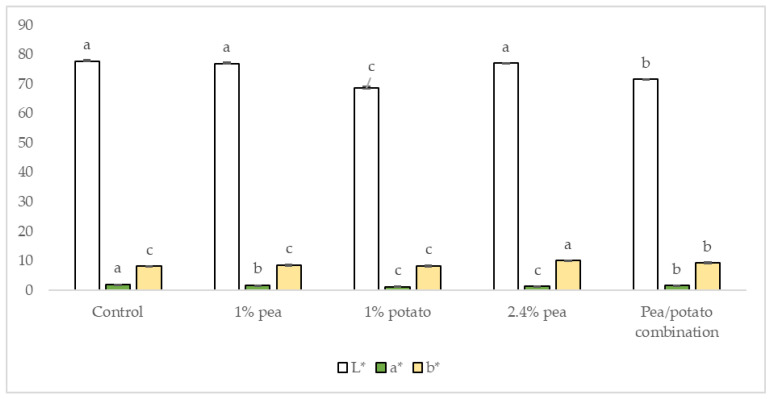
Colourimeter results; Lightness (L*) Green direction (reversed values demonstrating greenness) (a*) and yellow direction (b*). Data represent the means of three replicates ± standard deviations (*p*-value < 0.0001). Differing small letters represent sample significance from multiple comparisons as determined by Tukey’s honest significance difference (at *p* = 0.05). Green note was measured in minus values, converted to positives for clarity on graph.

The colourimeter results (Figure 5) showed that the potato protein-fortified products exhibited lower lightness than all others, with the 1% potato product being significantly lower than the combination, suggesting that the potato protein was significantly reducing the lightness. This may be resulting from the aggregation of particles that led to the increased particle size throughout the UHT process. Particle size has been found before to decrease the lightness in OMAs [39]. In terms of yellow direction, the 2.4% pea product was significantly higher than all other samples. The control sample had the highest lightness, and least yellow direction—although this was not significant, may suggest that protein fortification in general was decreasing the lightness and increasing the yellow colour.

### 3.6. Stability

**Figure 6:**
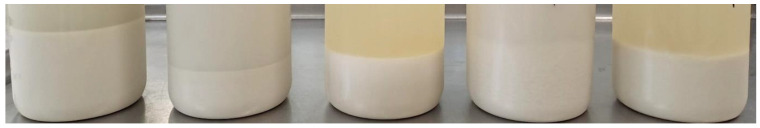
Image of samples after 3 days refrigeration, indicating stability. Left to right: control, 1% pea, 1% potato, 2.4% pea, and pea/potato combination.

Figure 6 shows an increased extent of separation after 72 h, in the samples containing potato protein. This may be a result of the increased particle size. Milk with lower particle size has previously been found to have an increased lightness in comparison to those with larger particles, as a result of light scattering [41]. However, it seems that the 1% pea and 2.4% pea samples may have a slightly reduced separation rate. It is possible that the pea protein isolate may provide some stabilising effect [25], potentially due to its emulsifying properties, with an emulsifying stability of 95.1-96.1% at pH 7 [42]. Emulsifying stability is the measurement of stability of an emulsion over a certain time, due to the magnitude of specific interactions such as electrostatic repulsion, and Van der Waals forces [18].

## 4. Discussion

The sensory data confirm that addition of pea protein results in a bitter and metallic taste, as well as an astringent aftertaste. This may be resulting from the non-volatile compounds; isoflavones which are associated with bitterness, phenolic acids which are associated with metallic taste, and astringency from the saponins [30].

From the volatile data, it was evident that the pea protein-fortified samples exhibited larger quantities of almost all volatile compounds recorded. This was reflected in the sensory results with a higher overall aroma intensity in this sample. The issues with off-flavours may be exacerbated by the UHT stage of production, with ultra-heat treatments having been found to greatly change the volatile aroma composition of pea protein-based beverages [43]. The colourimeter results also suggest that the pea protein may be increasing the yellow colour, which was also noticed within the panel with an increased perceived off-white colour. These results were all significantly different between the samples, suggesting that the 2.4% protein addition which is needed to match the 3.4% protein level in cow’s milk, would result in significant noticeable off-flavours and colours. An increase in stability however was found in the pea protein samples, with the 2.4% pea appearing to exhibit much less separation. This may be resulting from the emulsifying properties of pea protein [25]. However, it may be possible to develop methods to reduce off-flavours if the lipid degradation resulting in the volatile compounds responsible for this can be reduced. Aromatic properties of extracted protein can be influenced by the conditions of processing, including temperature, time, concentration, and pH—with pea protein extracted at pH 9 having been shown to exibit a lower beany flavour due to reduced lipoxygenase activity [44]. Breeding and cultivation of pea varieties with desired flavour properties may also be an option, as genotype can have a significant effect on 2/3-methyl-1-butanol, 2-pentylfuran and hexanal concentration [45]. It has also been suggested that conjugating pea protein with gum arabic could reduce the beany flavour [46].

1,3-Pentadiene was substantially higher in the pea/potato combination product than all others. 1,3 Pentadiene, may be produced by the conversion of sorbic acid, a widely used preservative in the food industry, through a decarboxylation mechanism in the presence of cinnamic acid [47]. Therefore, a possibility may be for each of the pea and potato proteins to contain one of these acids, with the combination resulting in this mechanism. However, neither were measured within this study to confirm.

The control product had significantly more froth/foam and the highest bubble size compared to all protein-fortified samples as concluded from the sensory results, suggesting the increased concentration of protein hindered the frothy/foaminess and bubble size. This may be a result of the proportion of oat flour in place of protein, as this would increase the concentration of starch, which has been shown to increase viscoelasticity and therefore foam strength [48]. Increased oat content would also increase the amount of avenacosides—oats’ unique saponins [49], which are known for their foaming properties [50]. The difference may also be related to the increased pH of the pea protein samples, with 2.4% pea at pH 7.53, in comparison to the control at 7.06, as pH has been shown to influence the stability of foams with protein–polysaccharide complexes [51]. Potentially additional unlisted ingredients from within the pea protein isolate may have affected the foaming, as presence of phospholipids, free fatty acids and partial glycerides have been shown to strongly impair the foaming of milk [52].

The 1% potato product had a significantly larger particle size than other samples. The increased particle size may have occurred during the UHT, which heats the product above 140 °C, as temperatures above 80 °C have previously been shown to damage hydrophobic interactions leading to a replacement of beta structures with alpha structures as temperatures increase [53], which has been described as stretching of the protein [23]. This larger particle size noticeably affected the sensory results, in terms of the powdery mouthfeel, mouthcoating effect and glass cling. This may also be responsible for the increased separation in this product. The large particle size in the potato protein also resulted in a reduced lightness in colour. It has been shown in a previous study [39] that a sensory panel detect this as a perceived “off-white” colour, which may be found to be unappealing.

The addition of both proteins, at all concentrations, contributed to an increased stale flavour, and off-white colour. The addition of proteins also resulted in a reduced single cream flavour, less froth/foam, and bubble size, whilst contributing to a floury flavour. It is not currently clear whether this would be considered a positive or negative effect, but may be an important consideration when protein additions are used in OMA production.

The significant sensory effects observed from both proteins may lead to a conclusion against their use in fortification of OMAs. However, research has shown that soy-based protein also exhibits strong aromas resulting from volatile compounds, in particular hexanal, including a “green” aroma [54], as well as a bitter taste resulting from the hydrophobicity of the protein [55]. Thermal treatment of soy milk can also result in the denaturation and aggregation of soy protein [56], potentially in a similar way as to what was observed in the potato protein in this study. Yet, soy milk sales remain the highest for all PBMAs, with an annual quantity of 265 million units sold on average between 2017-2019 [57], suggesting that these effects may not necessarily be a limiting factor for consumers.

Plant-based milks are of differing sensory standards to dairy in general, with OMAs exhibiting a lower whiteness index [5], and colour differences resulting from differences in particle size [6] when compared to cow’s milk. Off-flavours can result from the lipid degradation of unsaturated fatty acids and lipoxygenases, leading to the formation of n-hexanal and n-hexanol [2]. Therefore, it may be possible that some consumers expect a certain degree of off-colour and off-flavour in OMAs as a standard, and not be off-put by the additional effects of the protein fortification. To conclude this, further investigations into consumer liking with use of a consumer study, may be beneficial.

Methods of adjustment to the processing parameters may also provide a reduction in the sensory effects, without a need to replace the protein source. As a darker colour may be the result of high temperatures and pH during production leading to Maillard reactions, caramelisation, and oxidation of the phenolic compounds and lipids [58], methods such as ultra-high-pressure homogenisation (UHPH) may provide an alternative to high heat treatments. UHPH can lead to the lethality of microorganisms, whilst better preserving functional compounds including vitamins, polyphenols, and flavonoids [59]. Methods including washing, defatting, and milder processing conditions used throughout production could also contribute to a prevention of off-colour [60]. Multiple methods to reduce bitterness from pea protein, including the use of specific enzymes and organic compounds, have been successfully demonstrated to reduce bitterness as perceived by a sensory panel [61].

## 5. Conclusions

The results of this study demonstrated that the addition of pea and potato protein isolates at levels of just 1% were significantly affecting the sensory profile of OMA products. The effects were found to a greater extent at a level of 2.4% protein fortification, the approximate percentage required to match the levels of protein in cow’s milk. The changes detected are likely to be an issue when aiming to improve the protein content and composition of plant-based milks. A main contributor to undesired effects was the UHT stage of processing, with increased coagulation of particles within the potato protein samples, and increased lipid-derived volatiles from the pea protein samples, leading to sensory issues with the final product. Therefore, a key target of investigation into improving the sensory characteristics of the beverages may be the heat-processing stage of production.

As only one variant of each isolate was used for each protein type, it may be possible that other ingredient sources would elicit different effects. Additionally, there are many alternative sources of plant protein yet to be investigated in this context. Further investigation into alternative plant protein sources may need to be necessary, in order to continue to optimise the protein content in OMAs, whilst ensuring minimal effect on the sensory profile.

## Figures and Tables

**Table 1:** Protein addition to each sample to enable calculated final protein concentration (including oat protein from oats at 1%).

Sample Name ^a^	Protein Addition (g/100 g)	Final Calculated Protein Concentration
Control	0	1
1% Pea	1.2	2
2.4% Pea	3	3.4
1% Potato	1.068	2
Pea/potato Combination	0.6 (pea)/0.534 (potato)	2

^a^ The protein source for pea was an 80% protein isolate, and a 93.2% potato protein isolate for potato.

**Table 2:** Summary of attributes evaluated by the trained panel with reference and/or description used to confirm attribute definitions.

Modality	Attribute ^a^	Reference and/or Descriptor ^b^
Appearance	Off-white colour	D: amount of darkness or colour away from pure white
	Froth/foam	D: visual foam on surface of sample without stirring
	Bubble size	D: Visual perceived size of bubbles on surface of sample without stirring
	Glass cling	D: visual residue of sample on glass vial
Aroma	Overall intensity	D: strength of all aromas combined
	Sweet	Ref: Aqueous solution of sucrose
	Nutty	Ref: Blended mixed nuts
	Wet oats	Ref: Oats + cold water, soaked overnight
	Single cream	Ref: Single cream
	Malt	Ref: Barley malt extract
	Stale	Ref: Flaked ground almonds
	Brown bread	Ref: Sliced brown bread
	Floury	Ref: Plain flour in water
	Playdoh	D: Homemade playdoh
	Green-note	Ref: Sugar snap pea
	Potato starch	Ref: Cooked potato
	Off-note	D: Mouldy/musty
Taste	Bitter	Ref: Aqueous solution of quinine
	Sweet	Ref: Aqueous solution of sucrose
	Acid	Ref: Aqueous solution of citric acid
	Metallic	Ref: Iron sulphate 0.0036 g/L
Flavour	Stale	Ref: Flaked ground almonds
	Brown bread	Ref: Sliced brown bread
	Nutty	Ref: Blended mixed nuts
	Wet oats	Ref: Oats + cold water, soaked overnight
	Single cream	Ref: Single cream
	Malt	Ref: Barley malt extract
	Floury	Ref: Plain flour in water
	Green-note	Ref: Sugar snap pea
	Potato starch	Ref: Cooked potato
	Off-note	D: Mouldy/musty
Mouthfeel	Mouthcoating	D: Residue of sample in mouth
	Powdery	D: Grainy or large particle size
	Astringency	Ref: Tannic acid 0.2 g/L
	Body	D: Sensation of palate fullness and viscosity
Aftertaste	Bitter	Ref: Aqueous solution of quinine
	Metallic	Ref: Iron sulphate 0.0036 g/L
	Sweet	Ref: Aqueous solution of sucrose
	Single cream	Ref: Single cream
	Wet oats	Ref: Oats + cold water, soaked overnight
After-effects	Mouth coating	D: Residue of sample in mouth after swallowing
	Powdery	D: Grainy or large particles left in mouth after swallowing
	Astringency	Ref: Tannic acid 0.2 g/L
	Salivating	D: Saliva produced after swallowing

^a^ All anchors nil to extreme; ^b^ Ref = reference used; D = descriptor used to confirm attribute to panel.

**Table 3:** Mean panel scores for sensory attributes of the five samples.

Attributes	Mean Score (0–100) ^a^					Significance of Sample (*p*-Value) ^b^
	Control	1% Pea	1% Potato	2.4% Pea	Pea/Potato Combination	
**Aroma**						
Overall Intensity	41 ^b^	49.3 ^ab^	41.9 ^b^	57.7 ^a^	45.7 ^b^	<0.0001
Sweet	13.8	10.5	12.3	10	15.2	0.056
Wet Oat	29	25.4	25.8	30	33.9	0.23
Malt	0.5	1.4	4.3	0.4	4.4	0.30
Nutty	1	3.5	0.8	3.6	3.9	0.15
Stale	5.4	8.4	7	10.3	6.2	0.35
Single Cream	0	0	0.2	0.1	0	0.071
Brown Bread	4.3	10.2	12.7	9.7	9.3	0.22
Floury	11.6	14.7	16.7	18.9	13.3	0.39
Playdoh	15.5	20.9	9.6	21.6	13.2	0.12
Green Note	2.2	4.1	2	6.5	2.9	0.42
Potato Starch	4.2	5.8	11.2	4	8.6	0.19
Off-Note	3	6.2	1.3	5.8	0.8	0.16
**Appearance**						
Off-White colour	43.4 ^b^	48.8 ^ab^	51.1 ^ab^	55.5 ^a^	53.9 ^a^	0.0023
Glass Cling	30.6 ^b^	29.1 ^b^	46.2 ^a^	36.2 ^ab^	39.9 ^ab^	0.0004
Froth/foam	33.1 ^a^	8.2 ^b^	7.3 ^b^	5 ^b^	14 ^b^	<.0001
Bubble size	27.6 ^a^	6.6 ^b^	8 ^b^	3.3 ^b^	15.1 ^ab^	0.0001
**Taste**						
Sweet	18.9 ^a^	12.1 ^a^	15.5 ^a^	3.6 ^b^	14.8 ^a^	<.0001
Bitter	8.2 ^b^	17.1 ^ab^	8.1 ^b^	24.2 ^a^	12.5 ^b^	0.0002
Acid/Tang	0.8	1.4	0.8	4.7	0.8	0.082
Metallic	8.6 ^ab^	14.9 ^ab^	3.8 ^b^	17.1 ^a^	7.8 ^ab^	0.011
**Flavour**						
Malty	0.8	0.4	6	0.3	2.9	0.12
Wet oats	36.9	29.2	33	25.2	30.5	0.12
Nutty	6.5	4.1	3	2.7	4	0.45
Stale	3.3 ^b^	9.6 ^ab^	6.8 ^ab^	15.2 ^a^	8.4 ^ab^	0.021
Single cream	8.9 ^a^	1.4 ^b^	0.1 ^b^	0.1 ^b^	0 ^b^	0.0005
Brown Bread	4.7	5.5	4.3	5.6	5.2	0.98
Floury	5.2 ^b^	20.7 ^a^	19.9 ^a^	19.8 ^a^	20.9 ^a^	0.0041
Green Note	3.6	3	1.3	4.1	1.4	0.52
Potato Starch	1.6	5.2	8	7.9	9.7	0.39
Off-Note	3.1	7.1	2.2	11.1	2.8	0.11
**Mouthfeel**						
Mouthcoating	35.3 ^b^	31 ^b^	48.6 ^a^	37.4 ^ab^	41 ^ab^	0.0013
Body	37.8 ^ab^	29.9 ^ab^	29.2 ^b^	38.4 ^a^	35.5 ^ab^	0.012
Powdery	8.5 ^b^	16.8 ^b^	60 ^a^	12.5 ^b^	46.7 ^a^	<.0001
Astringency	12.1 ^b^	16.8 ^ab^	10.4 ^b^	22.7 ^a^	12.7 ^b^	0.0025
**Aftertaste**						
Bitter	4.3 ^c^	14.3 ^ab^	5.7 ^bc^	19.2 ^a^	9.3 ^bc^	0.0004
Metallic	9.5 ^ab^	16.5 ^a^	4.8 ^b^	17.2 ^a^	6.3 ^b^	0.0006
Sweet	11.1 ^a^	4.7 ^bc^	8.3 ^ab^	2.5 ^c^	8.9 ^ab^	0.0006
Wet oats	29.3	19.7	23.8	19.5	25.1	0.1
Single cream	5.2 ^a^	2.1 ^ab^	0 ^b^	0.1 ^b^	0 ^b^	0.0023
**After effects**						
Mouthcoating	17 ^b^	17.6 ^b^	29.7 ^a^	22.2 ^ab^	26.8 ^a^	0.0001
Powdery	6.2 ^b^	10.8 ^b^	45.8 ^a^	9.7 ^b^	29.3 ^a^	<0.0001
Astringent	10.1 ^b^	14.5 ^ab^	9.9 ^b^	19.8 ^a^	12.5 ^ab^	0.025
Salivating	19.8	18.2	17.6	19.3	17.7	0.97

^a^ Means not labelled with the same letters are significantly different (*p* < 0.05); means are from two replicate samples. ^b^ Probability of a significant difference between samples obtained from ANOVA.

## Data Availability

The original contributions presented in this study are included in this article; further inquiries can be directed to the corresponding author.
